# Burnout and Depressive Symptoms in Healthcare Professionals: A Cross-Sectional Study in Saudi Arabia

**DOI:** 10.3390/healthcare10122447

**Published:** 2022-12-05

**Authors:** Monira Alwhaibi, Tariq M. Alhawassi, Bander Balkhi, Noha Al Aloola, Aliyah A. Almomen, Abdulaziz Alhossan, Sarah Alyousif, Bana Almadi, Maryam Bin Essa, Khalid M. Kamal

**Affiliations:** 1 Department of Clinical Pharmacy, College of Pharmacy, King Saud University, Riyadh 11495, Saudi Arabia; 2 Medication Safety Research Chair, College of Pharmacy, King Saud University, Riyadh 11495, Saudi Arabia; 3 Department of Pharmaceutical Chemistry, College of Pharmacy, King Saud University, Riyadh 11495, Saudi Arabia; 4 Pharmaceutical Care Department, MNGHA, Riyadh 11426, Saudi Arabia; 5 College of Pharmacy, KSAH-HS, Riyadh 14611, Saudi Arabia; 6 King Abdullah International Medical Research Center, Riyadh 11481, Saudi Arabia; 7 Department of Pharmaceutical Systems and Policy, Health Sciences Center, School of Pharmacy, West Virginia University, Morgantown, WV 26506, USA

**Keywords:** burnout, depression, healthcare professionals, Maslach Burnout Inventory

## Abstract

Objectives. The study objectives were to examine the prevalence of burnout among healthcare professionals, analyze the association of depression and burnout among healthcare professionals, and explore the factors related to burnout. Methods. A prospective cross-sectional study using a validated questionnaire was conducted among healthcare professionals in a tertiary teaching hospital in Saudi Arabia’s central region. The Maslach Burnout Inventory (MBI) questionnaire was used to measure burnout through emotional exhaustion, depersonalization, and personal accomplishment. Descriptive and inferential statistics were carried out using SAS version 9.4. Results. The study sample was composed of 139 healthcare professionals. Around 48% of the study sample were nurses, 26% were physicians, 19% were pharmacists, and 6% were other healthcare professionals. About 61% screened positive for depression. Overall, one third of the participants had a high risk of burnout. Around 61.8% of the participants were in the high-risk group of the EE, 58.3% of the DP, and 41.0% of the PA subscales. Scores for the overall MBI were significantly different between various age groups, gender, those with social and financial responsibility, income, job titles, or years of experience. A higher risk of burnout in all subscales was observed among those with depression. Conclusions. A high risk of burnout was observed among healthcare professionals. The level of burnout was connected to workplace factors and the presence of depression. The burnout suffering among these healthcare professionals underlines the need to study further how to reduce the factors that contribute to burnout and the impact of interventions to reduce healthcare professionals’ burnout levels. The burnout scientific literature would benefit from further high-quality research with larger samples using longitudinal study designs to identify the causal risk factors.

## 1. Introduction

In the last decade, burnout syndrome has been progressively acknowledged as a significant problem affecting healthcare professionals. Burnout is a multifaceted syndrome, usually described by high emotional exhaustion, high depersonalization, and a low sense of personal accomplishment [1,2]. The exhaustion dimension is also labeled as feeling exhausted, a loss of energy, depletion, and fatigue. Depersonalization is described as negative or inappropriate attitudes towards patients, irritability, and withdrawal. The reduced personal accomplishment is defined as reduced productivity and an incapacity to cope [3]. Burnout is not recognized as a medical health condition in the International Classification of Diseases. Yet, emotional exhaustion has been considered the core symptom of burnout and the first symptom to develop in a worker in response to work stressors [4]. Thus, preventing exhaustion may prevent the development of other burnout symptoms. Multiple instruments have been developed in the scientific literature to evaluate burnout, such as the Maslach Burnout Inventory (MBI) [5], a self-administered questionnaire.

Many determinants of burnout have been identified in the literature. Some are related to work, and others are non-work-related factors [4]. For example, work-related factors among healthcare professionals resulting in burnout range from high stress at work, a high workload, number of hours worked [6,7], working shifts [8], and lack of organizational support [9,10]. Besides those determinants, the rapid global and national healthcare system changes in the payment models, electronic health records, and quality measures to deliver high-quality care, all impact how care is reimbursed, documented, and evaluated [11]. Because healthcare professionals are at the front of applying these required changes in response to high demands, and may overload to adapt to these changes, this could result in emotional exhaustion developing first, which could lead to detachment and negative reactions to the job (depersonalization). Burnout can be attributed to non-work-related factors such as individual dispositions (e.g., personality traits) that affect job stress experiences).

Burnout is a major concern globally across all healthcare professionals. It is counted as one of the major reasons for negative work-related outcomes (e.g., absenteeism, diminishing motivation, low performance, low personal achievement, and staff turnover) [9,12,13]. Also, burnout leads to lower productivity, poorer healthcare quality [14], lower patient safety [14], and higher medical errors [15,16,17], all of which, in turn, can reduce healthcare service quality [18]. Besides, studies have shown that burnout is associated with a negative impact on physical and mental health (i.e., emotional, psychological, and social wellbeing) [19,20], has adverse psychological effects such as depression [21,22], anxiety [6], obsessive–compulsive disorder [22], somatization [22], disturbed sleep, and fatigue.

Burnout syndrome among healthcare professionals has been reported worldwide. A physician-based study recognized that more than half of the US physicians met standards for burnout syndrome [23]. This study also reported burnout prevalence increased among US physicians to 54.4% in 2014 [23]. Studies documented a burnout rate of 44–56% in community pharmacists [6,24] and 61% in clinical pharmacists [25]. In the Middle East, the reported burnout prevalence range was between 40 and 60% [26]. A study comparing healthcare professionals reported a higher burnout among nurses [27]. Burnout is also common among medical faculties [28] and students in the healthcare field [29]. Burnout is more prevalent among females [9,30], married [7,31], and younger healthcare professionals as compared to their colleagues [7,32].

Despite the prevalence and effect of burnout on healthcare professionals in other nations, insufficient studies have assessed the healthcare professionals’ burnout and its associated factors in Saudi Arabia [31,32,33,34,35,36,37]. Thus, there is a need for the current study; to identify the prevalence and the probable reasons for burnout occurrence in healthcare professionals in Saudi Arabia.

## 2. Methods

### 2.1. Study Design and Setting

A cross-sectional survey was conducted in a tertiary teaching hospital (King Saud University Medical City) in Saudi Arabia’s central region. This hospital is one of the largest tertiary hospitals in Riyadh, Saudi Arabia, with a facility of more than 1000 beds that provides general and subspecialty medical services. The hospital patient population comprises mainly citizens from the Riyadh region and referred cases from the entire country. Approval by the Institutional review board (IRB) at KSUMC (IRB number: E-19-3667) was received.

### 2.2. Study Sample

Study participants comprised hospital healthcare professionals (physicians, pharmacists, nurses, and others). Students, technicians, and those on vacation/sick/maternity leave or attending professional training were excluded from the study.

### 2.3. Sample Size Calculation

The sample size was calculated using G*Power software for calculating the required number to conduct chi-square tests [38], where the alpha level was 0.05 and effect size = 0.5 for a confidence level of 95%. The minimum sample size was estimated to be 80 participants; however, more participants were recruited to ensure the sample size adequacy.

### 2.4. Procedures

#### 2.4.1. Questionnaire Development

The questionnaire was developed after an extensive literature review to identify existing instruments. An initial draft of the questionnaire was developed, consisting of three sections: (1) The first section collected data on socio-demographics, work-related training, and practice characteristics; (2) The second section included a depression scale; (3) The third section includes the Maslach Burnout Inventory (MBI) Scale. Subsequently, a group of investigators (*n* = 7) reviewed the survey and the instrument’s content and clarity.

#### 2.4.2. Validation of the Questionnaire

After IRB approval, the survey was pilot tested. Content validity was judged by conducting cognitive interviews among a purposive sample of healthcare professionals of diverse backgrounds (N = 12). Modifications to the survey were completed following the cognitive interview to clarify the meaning of one word for one of the items in the scale “I’ve become more callous (i.e., uncaring) toward people since I took this job”. Afterwards, the survey was discussed among the study investigators after those revisions. The survey was then uploaded to the online-based survey software Google Forms.

#### 2.4.3. Data Collection

A sample of healthcare professionals was invited to participate. Six declined to participate, and two participants did not complete one or two pages of the survey, which resulted in a total of 139 participants in this survey. Each eligible participant was requested to fill out an informed consent form before continuing to complete an anonymous online survey.

### 2.5. Measures

#### 2.5.1. Dependent Variable: Burnout

The Maslach Burnout Inventory (MBI) [5] is a self-reported, reliable, valid questionnaire used frequently to assess burnout levels among healthcare professionals [39]. The MBI is composed of 22 items divided into three subscales (Emotional Exhaustion (EE), Depersonalization (DP), and Personal Accomplishment (PA)). Participants were asked to read each item carefully and decide if they ever felt this way about their job. For example, EE items include, “I feel emotionally drained from my work”. Every item is rated on a seven-point Likert scale, based on the frequency upon which the respondents experience such feelings, from 0 for “never” to 6 for “every day” [40]. The aggregate scores on the subscales are used as cutoff points to identify different subgroups: EE (high > or =27, moderate 17–26 and low ≤16), DP (high ≥ 13, moderate 7–12 and low ≤ 6) and PA (high < or =21, moderate 38–22 and low ≥39). High scores on emotional exhaustion and depersonalization reflect higher degrees of burnout; low scores on personal accomplishment indicate higher degrees of burnout. Burnout severity is classified according to the subscales with a high score of EE and DP (low score of PA) (low: 1/3 dimension, moderate: 2/3 dimensions, and severe: 3/3 dimensions) [6].

#### 2.5.2. Independent Variables

The selection of the main independent variables was based on a conceptual framework developed by Dzeng et al. (2016) [41], which posits that a causal pathway from institutional culture climate to quality of care and subsequent burnout may affect patient and family outcomes, including quality of life. Workplace factors included the type of healthcare occupation (pharmacist, physician, nurse, others), working hours per week (≤40 h/week; >40 h/week), and the length of time that healthcare professionals have been working in the same job/setting/place (<one year, 1–2 years, 3–4 years. 5–10 years, >10 years).

Other independent variables included socio-demographic data (age, sex, marital status, nationality, number of children, smoking status, income level, financial responsibilities) and depression. Symptoms of depression were evaluated using the two-item Primary Care Evaluation of Mental Disorders (PRIME MD) validated scale described by Spitzer et al. [42]. A positive screening result meant answering “yes” to either one or both of the screening questions; a negative response to both questions is considered a negative result for depression. PRIME MD includes the following questions: “During the past month, have you often been bothered by feeling down, depressed, or hopeless?” and “During the past month, have you often been bothered by little interest or pleasure in doing things?” A positive screen for depression is defined as a “yes” response to either question. A positive response to the PRIME MD two-item instrument had a sensitivity of 96% and a specificity of 57% [43].

The existence of chronic conditions was evaluated by asking participants, “Within the past twelve months, has a doctor ever treated you for, or told you that you had, any chronic health condition?”.

### 2.6. Statistical Analysis

Mean ± standard deviation (SD) and frequency (%) were used for continuous and categorical variables. Chi-square tests were conducted on categorical variables. A *p*-value of less than 0.05 would be considered a priori as statistically significant. Statistical analyses were done using SAS version 9.4.

## 3. Results

### 3.1. Description of the Study Sample

The study sample was composed of 139 healthcare professionals (Table 1). The majority were men (56%) aged 40–49 (48.6%). About 74% were financially responsible for their family/parents. Around 48% of the study sample were nurses, 26% were physicians, 19% were pharmacists, and 6% were other healthcare professionals. About 76% of the healthcare professionals worked more than 40 h per week, and the majority had 3–10 years of work experience. About 20% had chronic health conditions, and 61% screened positive for depression.

### 3.2. Respondent Overall MBI Scores and Subscale Scores

Overall, around 32% had a high risk of burnout, 49% had a moderate risk, and 18% had a low risk (Table 2). The study sample mean scores for the EE, DP, and PA subscales were 31.6, 16.2, and 31.5, respectively. The mean scores for the EE and DP indicate a high level of EE and DP in our participants. However, the PA mean score indicates a medium level of PA in our participants.

Of the 139 participants, 86 (61.8%) were in the EE high-risk subscale group for burnout, 81 (58.3%) were in the high-risk subscale group for DP, and 51 (41.0%) were in the PA high-risk subscale group.

Scores for overall burnout level were significantly different between various age groups, gender, social and financial responsibility, income, occupation, or years of work experience.

The study population characteristics by the risk of burnout are summarized in Table 3. This study found a significantly higher percentage of high burnout risk among women than men (37.2% vs. 26.7%, *p*-value < 0.001). Participants with financial responsibility for parents/family had a higher risk of burnout than those without (40.2% vs. 27.2%, *p*-value < 0.001). Moreover, smokers had a higher risk of burnout than non-smokers (48.6% vs. 27.2%, *p*-value < 0.001). Looking closely at the monthly income, the group with a higher income had a lower percentage of a high risk of burnout compared to other groups (15.4%, *p*-value < 0.001). Nurses were predisposed to a higher risk of burnout than other professions (44.8%, *p*-value < 0.001). Also, a higher number of working hours was significantly related to an increased risk of burnout.

### 3.3. Respondent MBI Subscale Scores and Depression

When participants were grouped based on their risk of burnout as high, medium, or low for each subscale, a highly significant risk of burnout in all subscales was observed among those with depression (Table 4).

## 4. Discussion

In this study, approximately one third of participants had a high risk of burnout. A high risk of burnout was only considered to be present if a respondent had high EE, high DP, and low PA. A higher figure for burnout has been described in many countries in the Middle East [26,44]. A systematic review, including 138 published studies among healthcare professionals in the Middle East, reported a prevalence between 40 and 60% [26]. In this review, 18 studies in Saudi Arabia reported an overall burnout prevalence of between 18 to 88%. While most of these studies used the MBI assessment method, the variation can be attributed to the study setting and the type of healthcare occupation; some were among physicians and some among nurses.

The data confirm that burnout is a crucial concern among nurses, younger age groups, and women. Nurses appear more likely to have burnout than pharmacists and physicians. Data from the literature suggest that younger age groups [26,44] and nurses [26] suffer more from burnout than other age groups and other healthcare occupations. We observed a solid relationship between the risk of burnout and the participants’ gender; women had a higher risk of burnout. The sex difference is not unexpected in this study, as data from a systematic review reported that women seem to suffer more from burnout than men [26]. Our findings are compatible with published studies that demonstrate an increased risk of burnout among physicians, women, and individuals with depression; financial responsibility and occupation could be contributing factors to burnout [26].

There are plausible reasons to explain increasing burnout among healthcare professionals. For example, financial responsibility for parents/family and long working hours increase the risk of burnout. Depression was associated with a high risk of emotional exhaustion, depersonalization, and personal accomplishment. This relationship has been documented in a systematic review of 67 published studies [45]. Since the present study is cross-sectional, we are unable to determine whether burnout causes depression or depression causes burnout. There is an ongoing argument about the relationship between burnout and depression, whereby some studies contend that burnout is simply an atypical depressive disorder and overlap exists between burnout and depression (i.e., they are not distinct entities) and query the nosological added value of the burnout construct [46]. Indeed, some of the burnout symptoms (i.e., items on the MBI scale) appear to be similar to that of depression, such as loss of energy or fatigue, and cannot be overlooked. [47]. It has been widely documented that depressive and burnout symptoms frequently co-occur.

Our results highlight the need for future research on the mental health of healthcare professionals. Efforts should be made to screen healthcare professionals for signs and symptoms of depression. In addition, additional research is needed to describe how depression and burnout affect patient care. Lastly, interventional studies are necessary on methods to improve healthcare professionals’ mental health. Indeed, efforts to reduce burnout are primarily needed to provide patients with high-quality care. Studies reported that burnout is associated with medical errors [17]. This study’s findings will help open communication channels with stakeholders to discuss the level of healthcare professionals’ burnout and the need to design interventions to reduce it. Efforts to minimize burnout resulting from individual level factors need to integrate various approaches for healthcare professionals. There is growing evidence that mindfulness-based interventions among healthcare professionals can be beneficial by reducing stress and the burnout experienced and improving job satisfaction and patient health outcomes [48,49]. Mindfulness is commonly defined as the quality of awareness that occurs through purposely focusing on current moment experiences in an accepting manner [50]. A systematic review of thirteen studies highlighted that mindfulness-based stress reduction effectively reduced burnout [50]. In this review, the Mindfulness-based stress reduction (MBSR) program was an eight week group intervention of daily mindfulness practice via formal practices (e.g., body scan, meditation, mindful walking and yoga) and informal practices by mindfully engaging in typically mindless tasks (e.g., daily activities) [50]. Mindfulness-based stress reduction can improve burnout symptoms by enhancing wellbeing and handling stress.

The results of this study may have been predisposed to some limitations. First, we have used the MBI instrument to evaluate the burnout level; although it is considered a gold standard, three flaws with the MBI have been identified. These three include problems with the conceptualization, and technical and psychometric shortcomings, and it does not produce a single burnout score that can be dichotomized to distinguish between burned-out and non-burned-out cases [47,51]. Besides, recent studies have proposed that the dimension of ‘‘Personal Accomplishment’’ may not be part of the burnout concept, unlike the other two dimensions (i.e., emotional exhaustion and depersonalization). However, other existing burnout measurement instruments, such as the Copenhagen burnout inventory, also have shortcomings.

Second, our study cannot determine a causal relationship or the direction of this association. Third, unmeasured confounding variables could explain some of the associations observed. For example, fatigue, stress level, and personality traits could influence an individual’s level of burnout and make them more vulnerable. Besides, this study was performed in only one tertiary hospital in Saudi Arabia; therefore, the findings are not generalizable to all healthcare professionals in other healthcare centers and regions. Besides, we cannot exclude selection bias; participants included in this study may be depressed and have higher rates of chronic conditions than individuals seen in primary care settings.

## 5. Conclusions

In conclusion, burnout risk was high among healthcare professionals. The level of burnout was related to workplace factors and the presence of depression. These findings reinforce the need to reduce occupational stressors by building interventional programs for healthcare workers. Also, the suffering of burnout among healthcare professionals underlines the need to study how to minimize the predisposing factors contributing to burnout further. The burnout scientific literature would benefit from further high-quality research with larger samples using longitudinal study designs to identify the causal risk factors, and specifically burnout by occupation.

## Figures and Tables

**Table 1:** Characteristics of the Study Sample.

		N	%
Total		139	100.0
Age Group			
	20–39	24	17.6
	40–49	67	48.6
	50–60	47	33.8
Gender			
	Male	79	56.7
	Female	60	43.3
Nationality			
	Saudi	79	56.5
	Non-Saudi	60	43.5
Marital Status			
	Married	76	55.1
	Single/Divorced/Widowed	62	44.9
Number of children			
	None	57	41
	One	28	20.1
	More than One	38	27.3
	Missing	16	11.6
Financial responsibility for parents/family			
	Yes	103	73.4
	No	36	26.6
Smoking Status			
	Yes	35	25.4
	No	104	74.6
Exercise per week			
	0–1 h	77	55.4
	2–3 h	38	27.4
	4–5 h	15	10.7
	>6 h	9	6.5
Monthly Income			
	SR < 10,000	45	32.4
	SR 10,001–15,000	48	34.5
	SR 15,001–25,000	34	24.0
	SR > 25,000	12	9.1
Occupation			
	Physician	36	25.9
	Pharmacist	27	19.4
	Nurse	67	48.2
	Others	9	6.5
Number of hours work/week			
	≤40 h/week	33	23.7
	>40 h/week	106	76.3
Work Experience			
	<one year	19	13.6
	1–2 years	24	17.3
	3–4 years	43	30.9
	5–10 year	39	28.1
	>10 years	14	10.1
Chronic Health Conditions			
	Yes	28	20.1
	No	110	79.1
Depression			
	Yes	85	61.2
	No	54	38.8

Note: Study Sample Comprised 139 Healthcare Professionals. N: Number; SR: Saudi Riyals.

**Table 2:** Respondent Overall MBI Scores and Subscale Scores.

	Matric (Range)	Mean (SD) ^a^	Risk of Burnout N (%)		
			High	Moderate	Low
Burnout severity (Overall MBI)			45 (32.4)	67 (48.9)	26 (18.7)
I. Emotional Exhaustion ^b^					
	EE (0–54)	31.6 (15.1)	86 (61.8)	26 (18.7)	25 (17.9)
II. Depersonalization ^c^					
	DP (0–33)	16.2 (9.7)	81 (58.3)	24 (17.3)	33 (23.7)
III. Personal Accomplishment ^d^					
	PA (0–48)	31.5 (12.8)	31 (22.3)	50 (35.9)	57 (41.0)

MBI: Maslach Burnout Inventory; N: Number; SD: Standard Deviation. EE indicates Emotional Exhaustion; DP, Depersonalization; PA, Personal Accomplishment. Emotional Exhaustion (SUM) = Items (1, 2, 3,6, 8, 13, 14, 16, 20). Depersonalization (SUM) = Items (5, 10, 11, 15, 22). Personal Accomplishment (SUM) = Items (4, 7, 9, 12, 17, 18, 19, 21). ^a^ High scores on emotional exhaustion and depersonalization reflect higher degrees of burnout; low scores on personal accomplishment indicate higher degrees of burnout. ^b^ Emotional exhaustion scores range 0–16 (low), 17–26 (medium), 27 or higher (high); ^c^ Depersonalization scores range 0–6 (low), 7–12 (medium), 13 or higher (high); ^d^ Personal Accomplishment scores range 39 or higher (low), 22–38 (medium), 21 or lower (high). Burnout severity is classified according to the subscales with a high score of EE and DP (low score of PA) (low: 1/3 dimension, moderate: 2/3 dimensions and severe: 3/3 dimensions).

**Table 3:** Association Between Respondent Overall MBI Scores and Independent Variables.

		Risk of Burnout-MBI N (%)			*p*-Value
		High	Moderate	Low	
Age Group					0.039
	20–39	9 (47.4)	3 (15.8)	13 (36.8)	
	40–49	27 (40.3)	13 (19.4)	27 (40.3)	
	50–60	9 (19.1)	10 (21.3)	28 (59.7)	
Gender					<0.001
	Male	29 (37.2)	16 (20.5)	33 (42.3)	
	Female	16 (26.7)	10 (16.6)	34 (56.7)	
Number of children					<0.001
	None	22 (38.6)	6 (10.5)	29 (50.8)	
	One	10 (35.7)	7 (25.0)	11 (39.3)	
	More than One	12 (31.6)	7 (18.4)	19 (50.0)	
Financial responsibility for parents/family					<0.001
	Yes	41 (40.2)	16 (15.7)	45 (44.1)	
	No	4 (11.1)	10 (27.8)	22 (61.1)	
Smoking Status					<0.001
	Yes	17 (48.6)	5 (14.3)	13 (37.1)	
	No	28 (27.2)	21 (20.4)	54 (52.4)	
Monthly Income					<0.001
	SR < 10,000	12 (27.3)	8 (18.2)	25 (54.5)	
	SR 10,001–15,000	20 (41.7)	5 (10.4)	23 (47.9)	
	SR 15,001–25,000	11 (33.3)	12 (36.4)	10 (30.3)	
	SR > 25,000	2 (15.4)	1 (7.7)	17 (53.1)	
Occupation					<0.001
	Physician	9 (25.0)	7 (19.4)	20 (55.6)	
	Pharmacist	5 (19.2)	7 (26.9)	14 (53.8)	
	Nurse	30 (44.8)	9 (13.4)	28 (41.8)	
	Others	1 (11.1)	3 (33.3)	5 (55.6)	
Number of hours worked/week					<0.001
	≤40 h/week	1 (3.1)	8 (25)	23 (71.9)	
	>40 h/week	44 (41.5)	18 (16.9)	44 (41.5)	
Work Experience					<0.001
	<one year	3 (16.7)	5 (27.8)	10 (55.6)	
	1–2 years	6 (25)	3 (12.5)	15 (62.5)	
	3–4 years	20 (46.5)	7 (16.3)	16 (37.2)	
	5–10 year	12 (30.8)	9 (23.1)	18 (46.1)	
	>10 years	4 (32.4)	2 (14.3)	8 (57.1)	
Chronic Disease					<0.001
	Yes	6 (21.4)	6 (21.4)	16 (48.2)	
	No	39 (35.4)	20 (18.8)	51 (46.4)	

MBI indicates Maslach Burnout Inventory.

**Table 4:** Association between Respondent MBI Subscale Scores and Depression.

		Total	Overall Burnout MBI			*p*-Value
Depression						<0.001
	Yes	85	25 (29.4)	17 (20)	43 (50.6)	
	No	53	20 (37.7)	9 (16.9)	24 (45.3)	
			Risk of Burnout-MBI-EE N (%)			
			High	Moderate	Low	
Depression						<0.001
	Yes	85	50 (58.8)	20 (23.5)	14 (16.5)	
	No	53	36 (67.9)	6 (11.3)	11 (20.7)	
			Risk of Burnout-MBI-DP N (%)			
			High	Moderate	Low	
Depression						<0.001
	Yes	85	51 (60.0)	13 (15.3)	21 (24.7)	
	No	53	30 (56.6)	11 (20.7)	12 (22.6)	
			Risk of Burnout-MBI-PA N (%)			
			High	Moderate	Low	
Depression						<0.001
	Yes	85	21 (24.7)	31 (36.5)	33 (38.8)	
	No	53	10 (18.8)	19 (35.8)	24 (45.3)	

MBI-EE indicates Maslach Burnout Inventory-Emotional Exhaustion; MBI-DP, Maslach Burnout Inventory-Depersonalization; MBI-PA, Maslach Burnout Inventory-Personal Accomplishment.

## Data Availability

The datasets used and analyzed during the current study are available from the corresponding author upon reasonable request.

## References

[1] Perlman B., Hartman E.A. (1982). Burnout: Summary and future research. Hum. Relat..

[2] Marek T., Schaufeli W.B., Maslach C. (2017). Professional Burnout: Recent Developments in Theory and Research.

[3] Maslach C., Leiter M.P. (2016). Understanding the burnout experience: Recent research and its implications for psychiatry. World Psychiatry.

[4] Shoman Y., Rousson V., Bianchi R., Guseva Canu I. (2022). Holistic Assessment of Factors Associated with Exhaustion, the Main Symptom of Burnout: A Meta-Analysis of Longitudinal Studies. Int. J. Environ. Res. Public Health.

[5] Maslach C., Jackson S.E., Leiter M.P. (1996). MBI: Maslach Burnout Inventory.

[6] Balayssac D., Pereira B., Virot J., Collin A., Alapini D., Cuny D., Gagnaire J.-M., Authier N., Vennat B. (2017). Burnout, associated comorbidities and coping strategies in French community pharmacies—BOP study: A nationwide cross-sectional study. PLoS ONE.

[7] Chen K.-Y., Yang C.-M., Lien C.-H., Chiou H.-Y., Lin M.-R., Chang H.-R., Chiu W.-T. (2013). Burnout, job satisfaction, and medical malpractice among physicians. Int. J. Med. Sci..

[8] Wisetborisut A., Angkurawaranon C., Jiraporncharoen W., Uaphanthasath R., Wiwatanadate P. (2014). Shift work and burnout among health care workers. Occup. Med..

[9] Dugani S., Afari H., Hirschhorn L.R., Ratcliffe H., Veillard J., Martin G., Lagomarsino G., Basu L., Bitton A. (2018). Prevalence and factors associated with burnout among frontline primary health care providers in low-and middle-income countries: A systematic review. Gates Open Res..

[10] Griner P.F. (2013). Burnout in health care providers. Integr. Med..

[11] Almalki M., FitzGerald G., Clark M. (2011). Health Care System in Saudi Arabia: An overview. EMHJ-East. Mediterr. Health J..

[12] Felton J. (1998). Burnout as a clinical entity—Its importance in health care workers. Occup. Med..

[13] De Oliveira G.S., Ahmad S., Stock M.C., Harter R.L., Almeida M.D., Fitzgerald P.C., McCarthy R.J. (2011). High incidence of burnout in academic chairpersons of anesthesiology: Should we be taking better care of our leaders?. J. Am. Soc. Anesthesiol..

[14] Salyers M.P., Bonfils K.A., Luther L., Firmin R.L., White D.A., Adams E.L., Rollins A.L. (2017). The Relationship Between Professional Burnout and Quality and Safety in Healthcare: A Meta-Analysis. J. Gen. Intern. Med..

[15] West C.P., Huschka M.M., Novotny P.J., Sloan J.A., Kolars J.C., Habermann T.M., Shanafelt T.D. (2006). Association of perceived medical errors with resident distress and empathy: A prospective longitudinal study. JAMA.

[16] Tawfik D.S., Profit J., Morgenthaler T.I., Satele D.V., Sinsky C.A., Dyrbye L.N., Tutty M.A., West C.P., Shanafelt T.D. (2018). Physician burnout, well-being, and work unit safety grades in relationship to reported medical errors. Mayo Clinic Proceedings.

[17] Shanafelt T.D., Balch C.M., Bechamps G., Russell T., Dyrbye L., Satele D., Collicott P., Novotny P.J., Sloan J., Freischlag J. (2010). Burnout and medical errors among American surgeons. Ann. Surg..

[18] Hall L.H., Johnson J., Watt I., Tsipa A., O’Connor D.B. (2016). Healthcare staff wellbeing, burnout, and patient safety: A systematic review. PLoS ONE.

[19] Arrogante O., Aparicio-Zaldivar E. (2017). Burnout and health among critical care professionals: The mediational role of resilience. Intensive Crit. Care Nurs..

[20] Centers for Disease Control and Prevention About Mental Health. https://www.cdc.gov/mentalhealth/learn/index.htm.

[21] Huri M., Bağış N., Eren H., Umaroğlu M., Orhan K. (2016). Association between burnout and depressive symptoms among Turkish dentists. J. Dent. Sci..

[22] Vittori A., Marinangeli F., Bignami E.G., Simonini A., Vergallo A., Fiore G., Petrucci E., Cascella M., Pedone R. (2022). Analysis on Burnout, Job Conditions, Alexithymia, and Other Psychological Symptoms in a Sample of Italian Anesthesiologists and Intensivists, Assessed Just before the COVID-19 Pandemic: An AAROI-EMAC Study. Healthcare.

[23] Shanafelt T.D., Hasan O., Dyrbye L.N., Sinsky C., Satele D., Sloan J., West C.P. (2015). Changes in burnout and satisfaction with work-life balance in physicians and the general US working population between 2011 and 2014. Mayo Clinic Proceedings.

[24] Jocic D.D., Krajnovic D.M. (2014). State anxiety, stress and burnout syndrome among community pharmacists: Relation with pharmacists’ attitudes and beliefs. Indian J. Pharm. Edu. Res..

[25] Jones G.M., Roe N.A., Louden L., Tubbs C.R. (2017). Factors Associated With Burnout Among US Hospital Clinical Pharmacy Practitioners: Results of a Nationwide Pilot Survey. Hosp. Pharm..

[26] Chemali Z., Ezzeddine F.L., Gelaye B., Dossett M.L., Salameh J., Bizri M., Dubale B., Fricchione G. (2019). Burnout among healthcare providers in the complex environment of the Middle East: A systematic review. BMC Public Health.

[27] Olley B. (2003). A comparative study of burnout syndrome among health professionals in a Nigerian teaching hospital. Afr. J. Med. Med. Sci..

[28] El-Ibiary S.Y., Yam L., Lee K.C. (2017). Assessment of burnout and associated risk factors among pharmacy practice faculty in the United States. Am. J. Pharm. Educ..

[29] Silva R.G., Figueiredo-Braga M. (2018). The roles of empathy, attachment style, and burnout in pharmacy students’ academic satisfaction. Am. J. Pharm. Educ..

[30] Ashkar K., Romani M., Musharrafieh U., Chaaya M. (2010). Prevalence of burnout syndrome among medical residents: Experience of a developing country. Postgrad. Med. J..

[31] Al-Turki H.A., Al-Turki R.A., Al-Dardas H.A., Al-Gazal M.R., Al-Maghrabi G.H., Al-Enizi N.H., Ghareeb B.A. (2010). Burnout syndrome among multinational nurses working in Saudi Arabia. Ann. Afr. Med..

[32] Selaihem A. (2013). Prevalence of Burnout Amongst Physicians Working in Primary Care in Riyadh Military Hospital, Saudi Arabia. Int. J. Med. Sci. Public Health.

[33] Wajid S. (2018). Prevalence of Burnout among National Guard Health Affairs Physicians in Dammam, Saudi Arabia-A Cross-sectional Study. Asian J. Pharm. (AJP) Free Full Text Artic. Asian J Pharm..

[34] Aldrees T., Badri M., Islam T., Alqahtani K. (2015). Burnout among otolaryngology residents in Saudi Arabia: A multicenter study. J. Surg. Educ..

[35] Ghazi B., Himd B., Makhdoom Y.M., Habadi M.I., Ibrahim A. (2018). Pattern of Burnout Among Medical Interns in Jeddah. Int. Ann. Med..

[36] Al Sareai N., Al Khaldi Y., Mostafa O., Abdel Fattah M. (2013). Magnitude and Risk Factors for Burnout among Primary Health Care Physicians in Asir Province, Saudi Arabia. East Mediterr Health J..

[37] Elbarazi I., Loney T., Yousef S., Elias A. (2017). Prevalence of and factors associated with burnout among health care professionals in Arab countries: A systematic review. BMC Health Serv. Res..

[38] Faul F., Erdfelder E., Lang A.-G., Buchner A. (2007). G*Power 3: A flexible statistical power analysis program for the social, behavioral, and biomedical sciences. Behav. Res. Methods.

[39] Iorga M., Dondas C., Sztankovszky L.-Z., Antofie I. (2018). Burnout syndrome among hospital pharmacists in romania. Farmacia.

[40] Leiter C.M.S.E.J.M.P. (2018). Maslach Burnout Inventory: Manual.

[41] Dzeng E., Curtis J.R. (2018). Understanding ethical climate, moral distress, and burnout: A novel tool and a conceptual framework. BMJ Qual. Saf..

[42] Spitzer R.L., Kroenke K., Williams J.B.W., Group a.t.P.H.Q.P.C.S. (1999). Validation and Utility of a Self-report Version of PRIME-MDThe PHQ Primary Care Study. JAMA.

[43] Whooley M.A., Avins A.L., Miranda J., Browner W.S. (1997). Case-finding instruments for depression. Two questions are as good as many. J. Gen. Intern. Med..

[44] El-Menyar A., Ibrahim W.H., El Ansari W., Gomaa M., Sathian B., Hssain A.A., Wahlen B., Nabir S., Al-Thani H. (2020). Characteristics and predictors of burnout among healthcare professionals: A cross-sectional study in two tertiary hospitals. Postgrad. Med. J..

[45] Koutsimani P., Montgomery A., Georganta K. (2019). The Relationship Between Burnout, Depression, and Anxiety: A Systematic Review and Meta-Analysis. Front. Psychol..

[46] Bianchi R., Boffy C., Hingray C., Truchot D., Laurent E. (2013). Comparative symptomatology of burnout and depression. J. Health Psychol..

[47] Schaufeli W.B., Desart S., De Witte H. (2020). Burnout Assessment Tool (BAT)—Development, Validity, and Reliability. Int. J. Environ. Res. Public Health.

[48] Escuriex B.F., Labbé E.E. (2011). Health care providers’ mindfulness and treatment outcomes: A critical review of the research literature. Mindfulness.

[49] Irving J.A., Dobkin P.L., Park J. (2009). Cultivating mindfulness in health care professionals: A review of empirical studies of mindfulness-based stress reduction (MBSR). Complement. Ther. Clin. Pract..

[50] Kriakous S.A., Elliott K.A., Lamers C., Owen R. (2021). The Effectiveness of Mindfulness-Based Stress Reduction on the Psychological Functioning of Healthcare Professionals: A Systematic Review. Mindfulness.

[51] Kristensen T.S., Borritz M., Villadsen E., Christensen K.B. (2005). The Copenhagen Burnout Inventory: A new tool for the assessment of burnout. Work Stress.

